# Gaps in smiles and services: a cross-sectional study of dental caries in refugee-background children

**DOI:** 10.1186/1472-6831-15-10

**Published:** 2015-01-22

**Authors:** Alicia Quach, Ingrid L Laemmle-Ruff, Tatiana Polizzi, Georgia A Paxton

**Affiliations:** Department of General Medicine, The Royal Children’s Hospital, Melbourne, Australia; Department of Health, Office of the Chief Health Officer, Melbourne, Australia; Murdoch Children’s Research Institute, Melbourne, Australia

**Keywords:** Caries, Children, Dental services, Oral health, Refugee

## Abstract

**Background:**

Refugees are reported to experience high rates of dental disease, although there are limited data on refugee children. The aim of this study was to report on oral health in refugee-background children in Australia, and to assess their follow-up at dental services.

**Methods:**

Cross-sectional study of opportunistic oral health screening and subsequent dental service use in refugee-background children attending a refugee health clinic in Victoria, Australia, between November 2006 – November 2010.

**Results:**

350 patients (0 – 18 years, mean age 8 years 7 months) had oral health screening; 241 (68.9%) were born overseas, (176 Africa, 65 other countries) and 109 (31.1%) were born in Australia to African-background families. Parents were concerned about oral health in 65/341 (19.1%) children, with specific concern about caries in only 9/341 (2.6%). On assessment, 155/336 (46.1%) had visible caries and 178/345 (51.6%) had caries experience (dmft/DMFT > 0). Where parents were concerned about caries, they were likely to be present (positive predictive value = 100%), however absence of parent concern about caries was not reassuring (negative predictive value = 56.1%).

Compared to Australian-born children of African background; African-born children were more likely to be referred for further dental care (adjusted PR 1.33, 95% CI [1.02 – 1.73]), although there was no statistically significant difference in caries prevalence. African-born children were less likely to have caries compared to other overseas-born children (adjusted PR 0.73, 95% CI [0.58 – 0.93]). Overall 187/344 (54.4%) children were referred for further dental care; 91/124 (73.4%) attended any dental appointment. Attendance rates were 90% with a phone reminder system for appointments, attendance reduced when this system lapsed.

**Conclusions:**

Oral health is an important public health issue in refugee-background children, despite low levels of parent concern and very few parent reported caries. Routine direct oral health assessment is important in refugee-background children and co-ordinated health systems may help improve their attendance at dental services.

## Background

Poor oral health is linked with low socio-economic status and disadvantage [1]. Available population data suggest refugee-background children in Victoria are far more likely to live in poverty compared to Victorian children overall and face multiple barriers to accessing health services [2]. Refugees are reported to experience high rates of dental disease, although there are limited Australian data on adults [3–11] and none on refugee children. A 2006 review found the oral health status of adult refugees in Australia was worse than the Australian population overall, and worse than other risk groups, including Indigenous Australians [3]. The international literature suggests refugee children have poor oral health [12–18], although there is variability amongst subgroups and African-background children may have relatively better oral health status compared to other refugee groups [12]. Anecdotal information from providers suggests oral health problems, in particular caries experience, are emerging areas of need in refugee-background Australians.

The aim of this study was to provide local data on family/self reported oral health issues, dental assessment findings and dental service use in refugee-background children attending a refugee health clinic for medical review.

## Methods

The Royal Children’s Hospital (RCH) Immigrant Health Clinic (IHC) provides post-arrival refugee health screening and medical consultation/management for children of refugee background. The clinic does not receive referrals for oral health concerns. New patients/families attending the IHC were offered opportunistic oral health assessment and oral health promotion. The study population was therefore a convenience sample of children attending for medical review, over the period November 2006 to November 2010.

The same dental therapist assessed patients throughout the study period in a standard medical outpatient room, using an overhead lamp and disposable glass mirrors. Assessment findings were documented during the consultation on a dental screening proforma based on the World Health Organization (WHO) Oral Health Assessment Form [19]. Patients requiring ongoing dental care were referred to the Royal Dental Hospital (RDH) or local dental services. Patients over 18 years of age and patients on other visa types (i.e. family or skilled visas) were excluded from the analysis. Where patients had more than one oral health assessment during the study period, only data from the earliest assessment were included.

Information collected included: patient demographics, requirement for interpreter assistance, parent/self-reported oral health concerns and dietary risk factors, findings on inspection (dentition type (primary/mixed/secondary), visible caries, caries experience, severe caries) and management plans (referral to dental services, treatment needs). Visible caries was defined as the presence of a cavity, undermined enamel or softened floor or wall on a tooth surface, detected by the naked eye with the aid of a plane mouth mirror [19]. Caries experience was defined as the presence of decayed (d/D), missing (m/M) or filled (f/F) teeth as a result of dental caries, calculated as dmft/DMFT scores [19, 20]. Any caries experience was defined as dmft or DMFT score >0; severe caries was defined as: dmft ≥ 6 in children with primary dentition; dmft ≥ 6 and/or DMFT ≥ 2 in children with mixed dentition; and DMFT ≥ 6 in children with secondary dentition [21, 22].

Follow-up of attendance by patients referred to RDH was collated retrospectively using the RDH electronic patient database to extract information on any scheduled appointment(s) and attendance at any scheduled appointment(s).

Data were analysed using Excel, (Microsoft Corporation, Redmond, Washington, USA) and STATA version 11 (StataCorp LP, College Station, Texas, USA). Proportions were calculated for the overall study population and subgroups: overseas-born (total, African-born, other) and Australian-born children of refugee background. Comparisons between overseas-born and Australian-born were restricted to children of African origin, after preliminary data analysis revealed all Australian-born children were born to African-background families. Chi-square tests of independence and two sample t-tests were used to compare demographic characteristics between subgroups, and prevalence ratios (PR) and confidence intervals (CI) were calculated. Log binomial regression was used to calculate adjusted PRs. PRs for any visible caries, any caries experience, and any referral were adjusted for sex and dentition type. PRs for specific caries experience outcomes were restricted by detention type then adjusted by sex. Age was not included in the final regression model in order to avoid over-adjustment, due to the close correlation between dentition type and age (sensitivity analysis adjusting for sex, dentition type and age gave very similar results). Further, in terms of the accuracy of recorded age, it is not uncommon for birthdates to be recorded incorrectly in refugee children, although this information is rarely available on the initial visit [23]. Positive predictive values (PPV) and negative predictive values (NPV) were calculated for reported caries vs. caries detected on assessment and any reported oral health concerns vs. referral for further dental care. Missing results are reported, but were excluded from calculations.

Assessment findings were documented as part of routine clinical care, hence formal consent was not obtained. All data were de-identified for analysis and examined retrospectively; the risk to patient confidentiality was not beyond that of standard clinical care. Ethics approval was obtained from the Royal Children’s Hospital Human Research Ethics Committee (HREC reference number 31233B&C).

## Results

### Demographics

Oral health assessments were available for 350 eligible children aged 0 – 18 years for the period November 2006 to November 2010. There were 180 (51.4%) males and 170 (48.6%) females; the mean age was 8 years 7 months (range 8 months – 17 years 11 months). Overall, 241 children (68.9%) were born overseas and 109 (31.1%) were born in Australia to refugee-background parents. There were 25 countries of birth and 25 different languages recorded, and an interpreter was required for 221/350 (63.1%) attendances. Of the 241 overseas-born children, 176 (73.0%) were from African source countries and 65 (27.0%) were from Asia, the Middle East or the Pacific region. All 109 children born in Australia were of African origin. All children’s residential postcodes were in fluoridated water regions at the time of data collection [24]. Demographic details are shown in Table 1.

**Table 1:** **Demographic information by birth origin**

		Total (n = 350)	Overseas-born (n = 241)	Overseas-born African (n = 176)	Overseas-born other (n = 65)	Australian-born (n = 109)
**Gender**						
Female	Number (%)	170 (48.6)	121 (50.2)	89 (50.6)	32 (49.2)	49 (45.0)
Male		180 (51.4)	120 (49.8)	87 (49.4)	33 (51.8)	60 (56.0)
**Age (years)**	Mean (SD)	8.6 (4.4)	9.6 (4.4)	9.5 (4.4)	10.0 (4.6)	6.4 (3.5)
	Median (range)	8.6 (0.7-17.9)	9.7 (0.8-17.9)	9.5 (0.8-17.9)	10.2 (1.4-17.6)	5.6 (0.7-14.1)
**Country of Birth (Parent country of origin in Australian born)**	Number (%)	Australia 109 (31.1)	Sudan 76 (31.5)	Sudan 76 (43.2)	Burma 23 (35.4)	Somalia 79 (72.5)
		Sudan 76 (21.7)	Ethiopia 29 (12.0)	Ethiopia 29 (16.5)	Iraq 10 (15.4)	Sudan 12 (11.0)
		Ethiopia 29 (8.3)	Burma 23 (9.5)	Somalia 19 (10.8)	Thailand 9 (13.9)	Eritrea 12 (11.0)
		Burma 23 (6.6)	Somalia 19 (7.9)	Kenya 14 (8.0)	Afghanistan 5 (7.7)	Ethiopia 3 (2.8)
		Somalia 19 (5.4)	Kenya 14 (5.8)	Egypt 8 (4.5)	Iran/Malaysia both 4 (6.2)	Kenya 2 (1.8)
		Other 94 (26.9)	Other 80 (33.2)	Other 30 (17.0)	Other 10 (15.4)	Egypt 1 (0.9)
**Language spoken at home**	Number (%)	Somali 84 (24)	Dinka 60 (24.9)	Dinka 60 (34.1)	Karen 20 (30.8)	Somali 50 (45.9)
		Dinka 69 (19.7)	Somali 34 (14.1)	Somali 30 (17.1)	Chin 13 (20.0)	English 38 (34.9)
		English 66 (18.9)	English 28 (11.6)	English 26 (14.8)	Pashto 5 (7.7)	Dinka 9 (8.3)
		Arabic 30 (8.6)	Arabic 22 (9.1)	Arabic 19 (10.8)	Somali 4 (6.2)	Arabic 8 (7.3)
		Karen 20 (5.7)	Karen 20 (8.3)	Tigrinya 12 (6.8)	Arabic, Assyrian, Hakka, Iraqi - each 3 (4.6)	Tigre 2 (1.8)
		Other 81 (23.1)	Other 77 (32.0)	Other 29 (16.5)	Other 11 (16.9)	Other 2 (1.8)
**Interpreter required**	Number (%)	221 (63.1)	184 (76.4)	128 (72.7)	56 (86.2)	37 (33.9)

### Parent/self-reported concerns and dietary habits

Parent/self reported oral health concerns were present for 65/341 (19.1%) patients and dietary risk factors were reported by 78/342 (22.8%) (Table 2). The most frequent concerns were orthodontic/cosmetic, pain and caries.

**Table 2:** **Parent/self-reported oral health concerns and habits by birth origin**

		Total (n = 350)	Overseas-born (n = 241)	Overseas-born African (n = 176)	Overseas-born other (n = 65)	Australian-born (n = 109)
		Number (%)	Number (%)	Number (%)	Number (%)	Number (%)
**Oral health concern**	No concern	276 (80.9)	180 (77.3)	128 (75.3)	52 (82.5)	96 (88.9)
	Any concern	65 (19.1)	53 (22.7)	42 (24.7)	11 (17.6)	12 (11.1)
	Pain	20 (5.9)	20 (8.6)	15 (8.8)	5 (7.9)	0 (0)
	Hypersensitivity	2 (0.6)	2 (0.9)	2 (1.2)	0 (0)	0 (0)
	Trauma	2 (0.6)	1 (0.4)	0 (0)	1 (1.6)	1 (0.9)
	Gum disease	1 (0.3)	1 (0.4)	1 (0.6)	0 (0)	0 (0)
	Caries	9 (2.6)	3 (1.3)	1 (0.6)	2 (3.2)	6 (5.6)
	Orthodontic/cosmetic	21 (6.2)	18 (7.7)	16 (9.4)	2 (3.2)	3 (2.8)
	Extra teeth	3 (0.9)	3 (1.3)	3 (1.8)	1 (1.6)	2 (1.9)
	Other	8 (2.4)	6 (2.6)	5 (2.9)	0 (0)	0 (0)
	Missing	9	8	6	2	1
**Dietary habits**	Nil significant	264 (77.2)	181 (77.0)	134 (78.4)	47 (73.4)	83 (77.6)
	Any significant	78 (22.8)	54 (23.0)	37 (21.6)	17 (26.6)	24 (22.4)
	High sugar intake	72 (21.1)	49 (20.9)	36 (21.5)	13 (20.3)	23 (21.5)
	Nursing bottle > 2 yrs	6 (1.8)	5 (2.1)	1 (0.6)	4 (6.3)	1 (0.9)
	Missing	8	6	5	1	2

### Examination findings

Examination findings are shown in Table 3. Approximately equal proportions of children had primary, mixed and secondary dentition, 155/336 (46.1%) had visible caries and 178/345 (51.6%) had caries experience (dmft/DMFT > 0). Mean dmft/DMFT scores were relatively low in all sub-groups, but there was a wide range, with dmft scores of up to 13 in some children. Overseas-born children from other (non-African) countries consistently had the highest mean dmft/DMFT scores.

**Table 3:** **Examination findings by birth origin**

		Total (n = 350)	Overseas-born (n = 241)	Overseas-born African (n = 176)	Overseas-born other (n = 65)	Australian-born (n = 109)
		Number (%)	Number (%)	Number (%)	Number (%)	Number (%)
**Dentition**	Primary	113 (32.4)	56 (23.3)	39 (22.3)	17 (26.2)	57 (52.3)
	Mixed	127 (36.4)	85 (35.4)	67 (38.3)	18 (27.7)	42 (38.5)
	Secondary	109 (31.2)	99 (41.3)	69 (39.4)	30 (46.1)	10 (9.2)
	Missing data	1	1	1	0	0
**Visible caries**	No caries	181 (53.9)	110 (47.6)	88 (52.1)	22 (35.5)	71 (67.6)
	Any caries	155 (46.1)	121 (52.4)	81 (47.9)	40 (64.5)	34 (32.4)
	Enamel lesions	74 (22.0)	59 (25.5)	40 (23.7)	19 (30.6)	15 (14.3)
	1-3 carious teeth	35 (10.4)	26 (11.3)	20 (11.8)	6 (9.7)	9 (8.6)
	>3 carious teeth or pulp involvement	46 (13.7)	36 (15.7)	21 (12.4)	15 (24.2)	10 (9.5)
	Missing data	14	10	7	3	4
**Caries experience^**	*Primary dentition*					
	dmft = 0	72 (65.5)	32 (57.1)	25 (64.1)	7 (41.2)	40 (74.1)
	dmft >0	38 (34.5)	24 (42.9)	14 (35.9)	10 (58.8)	14 (25.9)
	*Mixed dentition*					
	dmft = 0 and DMFT = 0	42 (33.1)	24 (28.2)	23 (33.8)	1 (5.9)	18 (42.9)
	Any dmft or DMFT > 0	85 (66.9)	61 (71.8)	44 (65.7)	17 (94.4)	24 (57.1)
	*Secondary dentition*					
	DMFT = 0	52 (48.6)	45 (46.4)	32 (46.4)	13 (46.4)	7 (70)
	DMFT >0	55 (51.4)	52 (53.6)	37 (53.6)	15 (53.6)	3 (30)
	*Overall*					
	Any dmft >0 or DMFT > 0	178 (51.6)	137 (57.3)	95 (54.0)	42 (66.7)	41 (37.6)
**Mean (dmft/DMFT)**	*Primary dentition*					
	Mean dmft score (SD)	1.7 (3.0)	2.1 (3.1)	1.5 (2.6)	3.3 (3.8)	1.4 (2.9)
	Range	0-13	0-12	0-12	0-10	0-13
	*Mixed dentition*					
	Mean dmft score (SD)	2.5 (3.1)	2.9 (3.4)	2.5 (3.1)	4.4 (4.0)	1.6 (2.3)
	Range	0-12	0-12	0-12	0-11	0-8
	Mean DMFT score (SD)	0.3 (0.8)	0.3 (0.7)	0.3 (0.8)	0.4 (0.6)	0.3 (0.9)
	Range	0-5	0-4	0-4	0-2	0-5
	*Secondary dentition*					
	Mean DMFT score (SD)	1.6 (2.1)	1.7 (2.1)	1.6 (2.1)	1.8 (2.2)	0.7 (1.3)
	Range	0-9	0-9	0-9	0-8	0-4
**Severe caries**	*Primary dentition*					
	dmft 6+	15 (13.6)	9 (16.1)	3 (7.7)	6 (35.3)	6 (11.1)
	*Mixed dentition*					
	dmft 6+ or DMFT 2+	23 (18.1)	19 (22.4)	13 (19.4)	6 (33.3)	4 (9.5)
	*Secondary dentition*					
	DMFT 6+	5 (4.7)	5 (5.2)	4 (5.8)	1 (3.6)	0 (0)
	Any severe caries	43/345 (12.5)	33/231 (14.3)	20/176 (11.3)	13/63 (20.6)	10/109 (9.2)

^Missing data for dmft/DMFT scores: primary dentition – 3; mixed dentition – 0; secondary dentition – 2.

### Reported concern compared to objective findings

In the 65 children with parent/self reported oral health concerns, 54 were referred for review at dental services (PPV of parent concern for referral = 83.1%). However, in the 271 children with no parent/self reported concern and referral status recorded, 127 were referred for further review (NPV (lack of parent concern and not requiring referral) = 144/271 = 53.1%). Similarly, where parents reported caries they were likely to be present (PPV = 9/9 = 100%), however parents reported caries in only 9/341 (2.6%) children. Of the 319 children with no parent/self reported concern of caries and examination status recorded, 140 had caries (NPV (lack of parent concern and no caries) = 179/319 = 56.1%).

### Referrals and attendance at follow-up

In total 187/344 (54.4%) children were referred for further dental care (Table 4). Of those referred, 146 had their treatment needs recorded; 82/146 (56.2%) had complex oral health problems (needing pulpotomy, extraction or orthodontic care), 36/146 (24.7%) had restorative needs (simple direct fillings) and 28/146 (19.2%) had simple treatment needs (examination, scale/clean and oral hygiene instructions).

**Table 4:** **Referral rates for further dental care by birth origin**

	Total (n = 350)	Overseas-born (n = 241)	Overseas-born African (n = 176)	Overseas-born other (n = 65)	Australian-born (n = 109)	African-born vs Australian-born a) Crude PR, [95% C.I.] b) Adjusted PR, [95% C.I.]	African-born vs other overseas-born a) Crude PR, [95% C.I.] b) Adjusted PR, [95% C.I.]
	Number (%)	Number (%)	Number (%)	Number (%)	Number (%)		
**No referral**	157 (45.6)	88 (37.1)	67 (38.7)	21 (32.8)	69 (64.5)		
**Referral made (total)**	187 (54.4)	149 (61.8)	106 (61.3)	43 (67.2)	38 (35.5)	a) 1.73, [1.30 – 2.29] b) 1.33, [1.02 – 1.73]	a) 0.91, [0.74-1.12] b) Did not converge
**Referral made to RDH**	148 (43.0)	113 (47.7)	83 (48.0)	30 (46.9)	35 (32.7)		
**Referral made to other dental centre**	39 (11.3)	36 (15.2)	23 (13.3)	13 (20.3)	3 (2.8)		
**Missing data**	6	4	3	1	2		

Attendance information was available for 124/148 (83.7%) patients referred to RDH. Overall, 91/124 (73.4%) attended an appointment, 66/124 (53.2%) attended their first scheduled appointment, 33 patients (26.6%) did not attend any scheduled appointments. A phone reminder system coordinated through the referring clinic was implemented in 2008, with a concurrent attendance rate of 90% during that year. Attendance reduced after this system lapsed.

The median time from referral to first scheduled RDH appointment was 50 days (mean 106 days, range 2 – 1170 days). There was a substantial decrease in the waiting time between 2007 (median 274 days) and 2008 (median 45 days). The median waiting periods in 2009 and 2010 were 38 and 71 days respectively.

### Comparison between overseas-born and Australian-born children of African origin

The Australian-born group was significantly younger than the overseas-born African group (*t* = −6.2454, p < 0.001), reflecting the clinical observation that this group comprised younger siblings born to refugee-background families (Table 1). Consistent with this, dentition type differed between groups (χ^2^ = 40.669, p < 0.01), with a higher prevalence of primary dentition in Australian-born children (52.3% vs 22.3%). There was no significant difference in sex between groups (χ^2^ = 0.8495, p = 0.357). Comparisons between subgroups are shown in Table 5. The prevalence of visible caries was 47.9% (81/169) in African-born children compared to 32.4% (34/105) in the Australian-born African children; this was not statistically significant when adjusted for sex and dentition type (adjusted PR 1.35, 95% CI [0.97 – 1.88], p = 0.076). There was also no significant difference in caries experience or severe caries between the Australian-born and overseas-born African groups within primary and mixed dentition categories. Mean dmft/DMFT scores did not differ significantly between overseas and Australian-born children within primary (*t* = 0.2426, p = 0.809), mixed (*t* = −1.5364, p = 0.13 (dmft), *t =* 0.1074, p = 0.91 (DMFT)) and secondary dentition categories (*t = −*1.3419, p = 0.18). Compared to Australian-born children, overseas-born African children were more likely to be referred for further dental care (adjusted PR 1.33, 95% CI [1.02 – 1.73], p = 0.036).

**Table 5:** **Comparison of caries prevalence between groups**

Parameter		African born vs Australian born	African born vs Other overseas born
**Any visible caries**	Crude PR [95% C.I.]	1.48 [1.08 – 2.03]	0.74 [0.58 – 0.94]
	Adjusted PR [95% C.I.]	1.35 [0.97 – 1.88]	0.73 [0.58 – 0.93]
**Primary dentition**			
***dmft >0***	Crude PR [95% C.I.]	1.38 [0.75 – 2.56]	0.61 [0.34-1.08]
	Adjusted PR [95% C.I.]	1.35 [0.73 – 2.50]	0.54 [0.32-0.90]
***dmft 6+***	Crude PR [95% C.I.]	0.69 [0.18 – 2.60]	0.22 [0.06-0.77]
	Adjusted PR [95% C.I.]	0.66 [0.15 – 2.50]	0.17 [0.05-0.56]
***Mean dmft***	p-value^	0.809	0.045
**Mixed dentition**			
***dmft > 0 or DMFT > 0***	Crude PR [95% C.I.]	1.15 [0.84 – 1.58]	0.70 [0.57 – 0.85]
	Adjusted PR [95% C.I.]	1.14 [0.82 – 1.57]	Did not converge
***dmft 6+ or DMFT 2+***	Crude PR [95% C.I.]	2.04 [0.71 – 5.83]	0.58 [0.26-1.31]
	Adjusted PR [95% C.I.]	1.98 [0.69 -5.67]	0.57 [0.25-1.28]
***Mean dmft***	p-value^	0.13	0.033
***Mean DMFT***	p-value^	0.91	0.533
**Secondary dentition**			
***DMFT >0***	Crude PR [95% C.I.]	1.79 [0.68 – 4.72]	1.00 [0.67 – 1.51]
	Adjusted PR [95% C.I.]	1.79 [0.68 – 4.74]	1.00 [0.66-1.50]
***DMFT 6+***	Crude PR [95% C.I.]	N/A*	N/A*
	Adjusted PR [95% C.I.]	N/A	N/A
***Mean DMFT***	p-value^	0.18	0.367
**Overall: any dmft > 0 or DMFT > 0**	Crude PR [95% C.I.]	1.40 [1.07-1.85]	0.81 [0.65-1.02]
	Adjusted PR [95% C.I.]	1.24 [0.95 – 1.63]	Did not converge

^Two sample t-test of equal variance.

*A prevalence ratio for secondary dentition could not be calculated as there were no Australian-born children with severe caries and only one in the Other overseas born group.

### Comparison between overseas-born African children and overseas-born children from other countries

There was no significant difference in age (p = 0.22), dentition type (p = 0.44), or sex (p = 0.85) between the overseas-born children from Africa and overseas-born children from other countries. Overseas-born African children were less likely to have visible caries than children born in other source countries, (adjusted PR 0.73, 95% CI [0.58 – 0.93], p = 0.009). African children with primary dentition were less likely to have any caries experience (dmft > 0, adjusted PR 0.54, 95% CI [0.32 – 0.90], p = 0.017), had a lower mean dmft score (1.5 vs 3.3, p = 0.045) and were less likely to have severe caries (dmft ≥ 6, adjusted PR 0.17, 95% CI [0.05 – 0.56], p = 0.004) compared to children with primary dentition born in other countries. There was no significant difference in caries experience between these groups in children with mixed or secondary dentition.

## Discussion

This study provides the first data on oral health and dental service use in refugee-background children in Australia, and is one of few papers in the international literature examining oral health status in this group [12–18]. Over the time of this study, Australia’s annual Humanitarian intake was 13,500 people [25], with the majority arriving from Africa (Sudan, Somalia, Ethiopia), the Middle East (Iraq and Iran) and South Asia (Burma). This was reflected in participants being predominantly African-background.

Previous Australian studies of adult refugees have found higher rates of dental decay compared to the general Australian population, including other disadvantaged groups [3, 9, 10]. Other studies have shown that poor oral health is linked with low socio-economic status; lack of oral health education and insufficient levels of fluoride [1, 18, 26]. While these findings suggest refugee children are likely to have multiple risk factors for poor oral health, this (predominantly African) population had a similar prevalence of caries experience to Australian children overall. Recent Australian data showed caries prevalence for children aged 4 – 15 years was 41 - 67% with the highest prevalence in children aged eight years [21]. Our study population spanned a wider age range (0 – 18 years) and almost equal thirds of the group had primary, mixed and secondary dentition. The caries experience of the refugee group with mixed dentition was 66.9%; this sub-group may be more comparable to Australian data, suggesting the prevalence of caries in refugee-background children is toward the higher end of the Australian prevalence range. Further, almost all overseas-born children with mixed dentition from non-African source countries had caries experience (94.4%), suggesting this group may be of particular concern, although the diversity in areas of origin in the ‘other’ sub-group (Asia, Middle East and Pacific region) means comparisons should be interpreted with caution.

Other studies have found African children have fewer caries than other children [12, 27, 28]. There are several factors that may explain this observation. Firstly, fluoride has been shown to be protective against dental decay [29]. Many countries in the Horn of Africa have high levels of naturally occurring fluoride in the water supply, and fluorosis is noted in these areas [30, 31]. Secondly, the frequency of refined carbohydrate intake influences dental caries, through bacterial production of organic acids that demineralise/dissolve enamel [32]. Traditional African diets consist mainly of maize, millet, sorghum and rice, which are relatively low in refined sugars, and snacking between meals is not common in African populations. These factors are felt to be important in the lower caries prevalence associated with traditional African diets compared to that seen with Western diets [33]. Studies of sub-Saharan African migrants have found families retain traditional food practices [34, 35] and other oral hygiene practices (e.g. miswak) [35] that are associated with reduced caries.

In the international literature, the reported prevalence of caries in resettled refugee children ranges from 15.1% - 85% [12–14, 16–18]. Similar to the present study, five of these studies used convenience samples [12, 14, 16–18] with oral health screening performed alongside health screening. Only one study [13] used population sampling, and although this group had the lowest prevalence of caries (15.1%), it comprised predominantly immigrant children. Populations differed in terms of source countries (African [12, 17], European [12, 17], Asian [15], Chile and Middle East [18]), country of settlement (United States [12, 17], Canada [13], Netherlands [16], Sweden [18], Algeria [14]) and outcomes reported (caries experience [12–14, 16–18]; untreated decay [12]; oral hygiene [13, 16]; gingivitis [13]; fluorosis [14, 16]).

In this study, Australian-born African children had fewer caries than their older African-born siblings. The difference in mean age between the two groups (6.4 years vs 9.5 years) is likely to be a contributing factor to this observation. Older children have had longer cumulative exposure to risk factors for dental caries, including exposure to dietary sugars and micro-organisms, and poor oral hygiene practices. Migration to, and length of residence in a developed country may confer additional benefits alongside any protective factors related to ethnicity. Children from developing countries, especially those from areas of conflict or refugee camps, typically have very limited access to dental care [36]. Oral health may not be a high priority and caries may be left untreated, with people seeking dental treatment only when they have unbearable pain or require an extraction [10, 37]. In contrast, Australian children and their families receive oral health promotion during the pre-school and primary school years and there are a variety of programs supporting access to dental care. This cross-sectional study does not allow analysis of these factors, and a prospective cohort study is required to assess the impact of migration to a developed country on oral health over time.

Although parent/self reported oral health issues were infrequent, a significant proportion of children had abnormal findings on assessment, and over half were referred for further treatment; with a low negative predictive value for parent/self report. This suggests that in order to establish an accurate understanding of oral health problems in this group, history alone is likely to be inadequate, and direct assessment is important.

Guidelines for post-arrival refugee health assessments include oral health screening and health promotion in addition to screening for nutritional deficiencies and communicable diseases [6, 38]. Incorporating oral health screening and health promotion during post-arrival refugee health assessments was feasible and well received by families.

For newly arrived refugees in Australia, access to public dental health services may be limited. Davidson et al. (2007) reported variable costs for dental services and significant waiting periods, ranging from 13 to 58 months [5]. A more recent qualitative study of refugee-background families with preschool aged children examined families’ knowledge of oral health, experience of accessing dental services, and barriers and enablers to achieving adequate oral health [37]. This study found that families’ past experiences and resettlement issues were barriers to achieving good oral health, and further identified challenges accessing dental services, including long wait periods and lack of interpreting services. Despite RDH providing a free service to refugees and asylum seekers [39], routinely working with interpreters, and having a relatively short median wait time of 50 days, only 73.4% of referred patients were recorded as attending an appointment at the RDH. This proportion reduced further (to 61.5% or 91/148) when including patients where no scheduling information was available.

There are multiple reasons why refugee-background famillies fail to attend health or dental appointments. These include competing resettlement priorities, administrative issues, language barriers, lack of transport, traditional health beliefs or misunderstandings, and previous negative health service experiences [37, 40]. Administrative issues include failure to capture accurate demographic details (phone number, address) in a population that are frequently mobile in the early settlement period, and the routine practice of sending appointment letters in English (regardless of non-English speaking background or English print literacy). Our health service has used a phone reminder system since 2005, and maintains annual attendance rates above 85%, despite most patients requiring interpreter assistance. The observation that attendance at dental appointments improved with the use of a phone reminder system by the referring clinic in 2008 is important, offering a strategy to improve systems efficiency and facilitate attendance to dental services by patients with low English (or majority language) proficiency. The reduction in attendance after this system lapsed is also noteworthy, suggesting active surveillance is required.

There are several limitations to this study. Firstly, the study population was a convenience sample from a busy outpatient refugee medical service. However, previous studies have also used convenience samples from refugee camps [14] and refugee resettlement health screening programs [12, 16–18], reflecting the challenges in obtaining systematic data in this group. Patients attending the IHC were seen in medical consultation rooms, without routine dental equipment, which may have resulted in under-ascertainment of caries. Dental screening was performed by a single clinician, and while it was likely that assessment was consistent over time, no examiner calibration was performed prior to the study to ensure intra-examiner reliability and examination findings were not confirmed by a second observer. Information on oral health practice (including tooth brushing), an important protective factor for caries, was not available. Similarly, duration of residence in Australia, parent income, and parent education were not recorded routinely for overseas-born children, limiting examination of any protective effect of living in Australia, and restricting adjustment for these variables. The exclusion of (limited) missing data from analysis may have affected comparisons between subgroups. Performing a cross-sectional study only allowed for assessment of oral health at a point in time, which may have resulted in over or under-estimation of true oral health status. Finally, the study population was predominantly African, meaning results are not generaliseable to refugee children from other source countries. However, systematic data collection in refugee-background children remains challenging, and in the absence of other data, this study provides important information on oral health screening in refugee-background children, and highlights some of the challenges of working with this population.

## Conclusions

In this study, the prevalence of caries in (predominantly African) refugee-background children was similar to the caries prevalence in Australian children. Limited data suggest a higher prevalence of caries in refugee children from non-African source countries. Despite caries being common, parent concern about oral health was uncommon, and direct assessment of oral health is important in refugee children. Over half of this study group required further assessment at dental services, however attendance at follow-up was poor, despite comparatively short waiting periods and appointment reminders. A coordinated reminder system between health and dental services appeared to work well initially, suggesting this may be a way to improve attendance. This study provides the first Australian caries prevalence and service use data in refugee-background children, to inform public health policy and program development directed to this group.
